# Effects of a Minimal-Dose, Short-Duration Core Training Program on Core Function and Athletic Performance in Elite Female University Ice Hockey Players in Japan: A Case Series

**DOI:** 10.7759/cureus.114246

**Published:** 2026-08-10

**Authors:** Haruna Yoneyama, Shungo Fujii, Shouta Kaneko, Toshihiro Watanabe

**Affiliations:** 1 Health and Nutrition, Hokkaido Bunkyo University, Eniwa, JPN; 2 Nutrition, Hokkaido Bunkyo University, Eniwa, JPN; 3 Occupational Therapy, Hokkaido Bunkyo University, Eniwa, JPN; 4 Biochemistry, Hokkaido Bunkyo University, Eniwa, JPN

**Keywords:** core function, core training, exercise, ice hockey, sport training

## Abstract

Female university ice hockey players in Japan often have limited opportunities to perform additional conditioning because of demanding academic schedules and regular team training. Consequently, brief and practical exercise programs that can be incorporated into daily routines are needed. We report a prospective case series evaluating the feasibility and potential effectiveness of a minimal-dose core training program consisting of a 30-second prone static plank performed three times per week.

Three elite female university ice hockey players (one in her late teens and two in their early 20s), all with youth national team experience and competing in Japan's highest-level women's league, participated in this 24-week prospective case series conducted using a BAB single-case experimental design. Participants completed an initial eight-week intervention period, an eight-week withdrawal period, and a second eight-week intervention period while continuing their usual team training.

Core function was evaluated using the Sahrmann Core Stability Test (SCST) and Seated Side Tapping Test (SST), athletic performance was assessed using the sit-up test and 40-yard dash, and body composition was measured by bioelectrical impedance analysis. Improvements in SCST, SST, and sit-up performance were consistently observed during the intervention phases, whereas performance plateaued or declined during the withdrawal phase. Sprint performance and body composition changed minimally throughout the study. All participants reported excellent adherence to the program without interference with university life and described increased body awareness and motivation toward training.

This case series suggests that an extremely brief, high-frequency core training program may improve core function and muscular endurance through neuromuscular adaptation rather than muscle hypertrophy. Although the findings are limited by the small number of participants, this practical intervention may represent a feasible conditioning strategy for elite student-athletes with limited training time.

## Introduction

In Japan, men's ice hockey has a long history, with its professional league established in 1966 and its inclusion as an official Olympic sport since 1936. In contrast, the history of women's ice hockey is relatively short. It was first recognized as an official Olympic sport at the 1998 Nagano Winter Olympics. However, the sport has not been professionalized in Japan to the extent that athletes can make a living solely from playing ice hockey [1].

Consequently, female ice hockey players in Japan typically belong to amateur teams supported by local clubs or corporations. These teams include players ranging from junior high school students to working adults. Therefore, many athletes must balance competitive sport with academic studies or occupational responsibilities. Previous dual-career research has demonstrated that student-athletes commonly experience substantial time constraints and difficulties balancing educational and athletic demands, highlighting the importance of practical and time-efficient training strategies.

Because of limited ice-rink availability in Japan, on-ice practice is often scheduled during evening hours and typically lasts approximately 90 minutes. Furthermore, systematic foundational strength and conditioning programs are not universally implemented, and in-season conditioning frequently depends on the individual athlete's initiative.

This environment creates a strong demand for efficient training methods that can enhance physical function within a limited timeframe. A considerable proportion of elite female players in Japan, including those selected for national teams, are university students who must maintain a high level of athletic performance while simultaneously managing their academic responsibilities [2].

While the Japanese women's national team has achieved some success in international competitions, there is still room for improvement to reach the world's top level. Enhancing individual player performance is essential for further progress, and strengthening core function is considered a critical component. Core function refers to the ability of the trunk musculature to stabilize the spine and pelvis during movement and force transmission. Efficient core stabilization contributes to postural control, balance, and coordinated limb movement, all of which are essential for athletic performance in ice hockey. These functions are directly linked to key performance elements in ice hockey, such as skating stability, stickhandling precision, and postural control during body contact [3,4].

Previous studies have suggested that even low-volume exercise interventions may improve neuromuscular function and trunk stability when performed consistently [5,6]. Plank-based exercises have been reported to enhance trunk muscle activation and motor control without necessarily inducing substantial hypertrophic adaptations [7]. Because elite athletes often already possess relatively high baseline muscle strength and conditioning levels, subtle neuromuscular adaptations rather than large structural changes may be more relevant to performance enhancement [8]. Therefore, we hypothesized that a short-duration isometric intervention, such as a 30-second prone plank, might be sufficient to improve core-related function in this population.

Given this background, we implemented a short-duration core training program consisting of a 30-second prone static plank performed three times per week [5]. The static prone plank was selected because it promotes sustained activation of deep trunk musculature and postural stabilization while requiring minimal equipment and time commitment. Such static trunk stabilization may be particularly relevant for ice hockey athletes, who require efficient force transmission and trunk control during skating and body contact situations. A single-case experimental design with a BAB structure was adopted to evaluate intervention-related changes within individuals while maintaining ecological validity during the competitive season [9]. This design allowed repeated observation of changes across intervention and non-intervention phases in elite athletes under real-world training conditions.

This study targeted elite female university ice hockey players, specifically those with youth national team experience who were currently competing in Japan's top-tier women's league, to examine the effects of a brief core training program on core function, athletic performance, and body composition.

## Case presentation

Study design

This prospective case series was conducted at Hokkaido Bunkyo University, Japan, during the competitive season between December 2023 and June 2024. Consecutive recruitment was performed from members of the university women's ice hockey team who met the eligibility criteria.

The inclusion criteria were female university ice hockey players who had previous youth national team experience, were currently competing in Japan's highest-level women's ice hockey league, had more than 10 years of competitive experience, and had no musculoskeletal injuries that would prevent participation in training. Players with acute orthopedic injuries, neurological disorders, or medical conditions affecting exercise performance were excluded.

The study protocol was approved by the institutional ethics committee (Approval No. 05010), and written informed consent was obtained from all participants.

Baseline clinical characteristics

All participants were healthy elite athletes participating in regular university and team training. None had current low back pain, lower-extremity injury, neurological disease, or other medical conditions requiring treatment during the study period. Because the participants faced considerable academic commitments in addition to regular team practice, they reported limited opportunities for additional conditioning. Therefore, a brief core training program requiring less than one minute per session was introduced as a practical conditioning strategy rather than as treatment for a specific medical condition. Baseline physical examination confirmed that all participants were medically fit for sports participation.

Case 1

Participant A was a female university ice hockey player in her late teens (height 169 cm, body weight 61 kg, BMI 21.4 kg/m²) with 14 years of competitive experience and previous youth national team participation. She had no history of recent musculoskeletal injury or neurological disease.

Case 2

Participant B was a female university ice hockey player in her early twenties (height 158 cm, body weight 58.8 kg, BMI 23.6 kg/m²) with 14 years of competitive experience. She had no significant medical history or current orthopedic complaints. Pre-intervention assessment confirmed eligibility for participation.

Case 3

Participant C was a female university ice hockey player in her early 20s (height 159 cm, body weight 60.9 kg, BMI 24.1 kg/m²) with 15 years of competitive experience. She was free from injury throughout the study period and completed all intervention sessions.

Intervention

**Figure 1:**
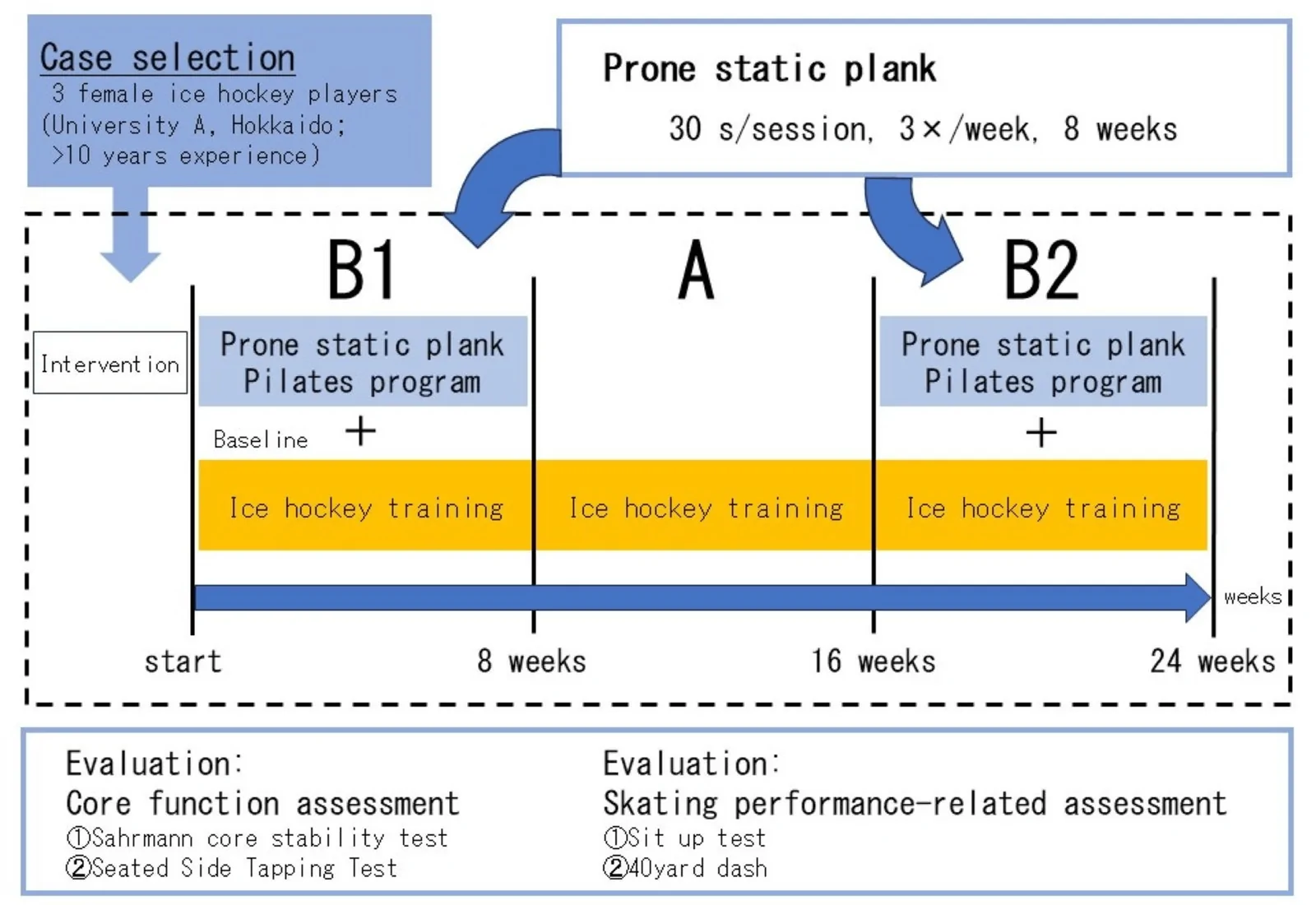
BAB-type single-case study design

Participants completed a prospective BAB single-case experimental protocol consisting of an initial eight-week intervention phase (B1), an eight-week withdrawal phase (A), and a second eight-week intervention phase (B2). A BAB design was selected because the participants were elite athletes during the competitive season, making an initial baseline phase impractical while allowing each participant to serve as her own control [9]. Throughout the study period, participants continued their regular team training, consisting of approximately three on-ice practice sessions (90 min/session) and three off-ice conditioning sessions (60 min/session) per week. No major changes were made to the overall training program during the study period. During the intervention phases, participants performed a single 30-second prone static plank exercise three times per week (Figure 1). This isometric bodyweight exercise was selected because it effectively activates the deep trunk musculature, requires no special equipment, and can be completed in less than one minute, making it highly feasible for time-constrained student-athletes [7]. Initial instruction was provided by a certified Pilates instructor. Participants were instructed to maintain a neutral spine, slight posterior pelvic tilt, activation of the deep abdominal muscles, appropriate scapular position, and controlled breathing. Exercise performance was monitored through video submissions, and individualized feedback was provided when necessary.

Outcome assessment

Outcome measures included the Sahrmann Core Stability Test (SCST) [10,11], the Seated Side Tapping Test (SST) [12], the sit-up test [4], the 40-yard dash [4], body composition assessed by bioelectrical impedance analysis (InBody S10) [13], and a post-intervention questionnaire.

For the analysis of intervention-related changes, visual inspection of the time-series data was conducted, including level, trend, variability, immediacy of effect, and consistency across phases. Regression analyses were conducted to quantify linear trends within each phase of the BAB design. The slope coefficient was used to describe the direction and magnitude of change over time. Visual analysis, regression analysis, and Non-overlap of All Pairs (NAP) were performed to evaluate intervention-related changes across the BAB phases (Table 1).

**Table 1:** Summary of visual analysis criteria and NAP outcomes SCST: Sahrmann Core Stability Test; SST: Seated Side Tapping Test; NAP: Non-overlap of All Pairs

Outcome	Level	Trend	Variability	Immediacy of Effect	Consistency	NAP
SCST	Improvement or maintenance of maximal score during intervention phases	Positive	Low	Present in Participants B and C	High	Low-Moderate (ceiling effect observed)
SST	Reduced completion time during intervention phases	Negative	Low	Present following re-intervention	High	Small-Moderate
Sit-up Test	Increased repetitions during intervention phases	Positive	Low	Present	High	Not calculated
40-yard Dash	Minimal change	Flat	Low	Absent	High	Not calculated

Result

Core Function

Core function was evaluated using the SCST and the SST.

**Figure 2:**
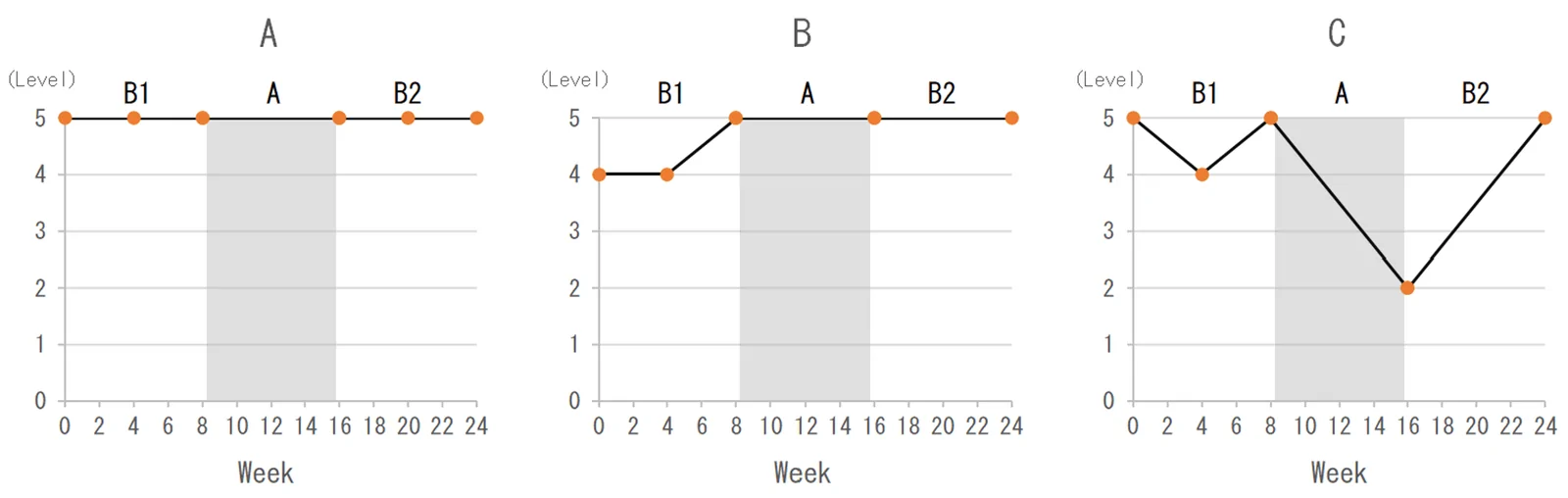
Sahrmann Core Stability Test (SCST) outcomes: participants A, B, and C Changes in SCST level during the intervention period.

On the SCST, trunk stability was evaluated using a pressure biofeedback unit positioned beneath the lower lumbar region. Participants performed standardized lower-limb movements while maintaining a baseline pressure of 40 mmHg. Advancement to the next level was permitted when pressure changes remained within ±10 mmHg. Scores ranged from Level 0 to Level 5, with the highest successfully completed level recorded as the test score. Higher scores indicated better trunk stability [10]. Participant A maintained the maximum score of Level 5 throughout the study period. Participant B improved from Level 4 at baseline to Level 5 during the first intervention phase (B1) and maintained this level thereafter. Participant C temporarily declined from Level 5 to Level 2 during the withdrawal phase (A) but recovered to Level 5 during the second intervention phase (B2). At the end of the study, all participants reached Level 5 (Figure 2).

**Figure 3:**
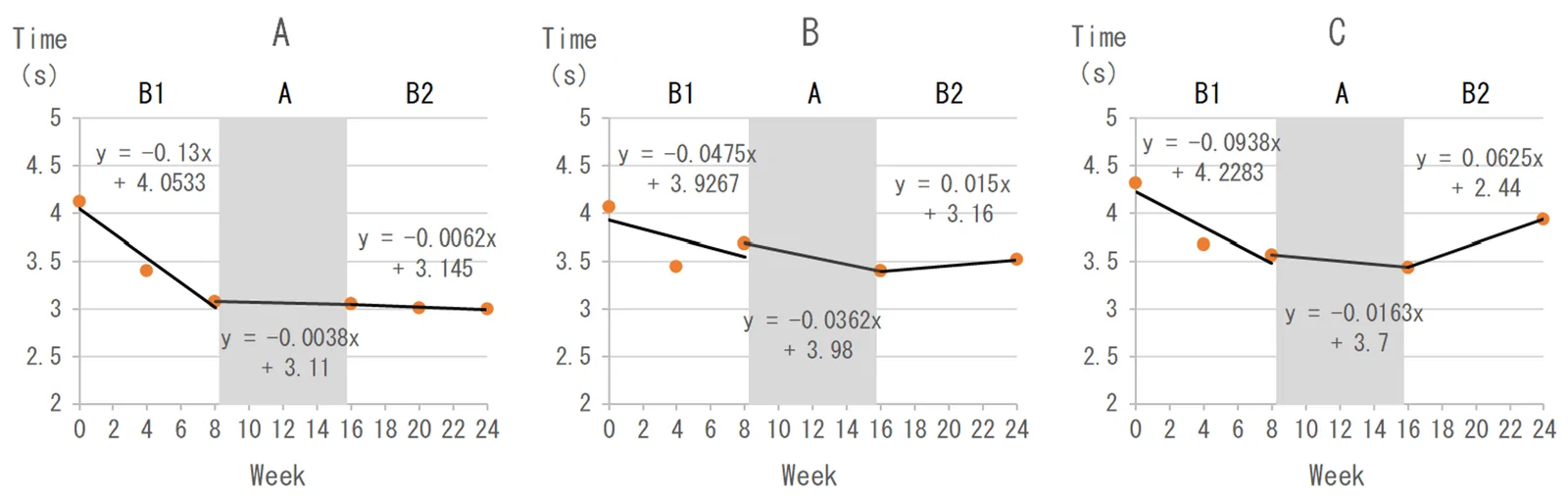
Seated Side Tapping Test (SST) outcomes: participants A, B, and C Changes in SST level during the intervention period. Linear regression lines were fitted separately for each phase of the BAB design, and the corresponding regression equations are shown.

On the SST, performance was evaluated as the time required to complete 10 alternating side taps [12]. Performance improved during both intervention phases in all participants. Participant A improved from 4.12 s at baseline to 3.00 s at the end of B2. Participant B improved from 4.07 s to 3.52 s, and Participant C improved from 4.32 s to 3.94 s. In all participants, performance plateaued or deteriorated during the withdrawal phase and improved again following reintroduction of the intervention. Regression analysis demonstrated negative slopes during the intervention phases, indicating progressive improvements in SST performance. For Participant A, the slope coefficients were β = -0.144 during B1 (R² = 0.922) and β = -0.021 during B2 (R² = 0.706). Participant B demonstrated a slope of β = -0.025 during B1 (R² = 0.110), whereas Participant C demonstrated a slope of β = -0.135 during B1 (R² = 0.821). Because each phase contained only a limited number of observations, these regression analyses should be interpreted descriptively rather than as inferential evidence (Figure 3).

Athletic Performance

Performance was evaluated using the sit-up test and the 40-yard dash, following previously described methods [3,4].

**Figure 4:**
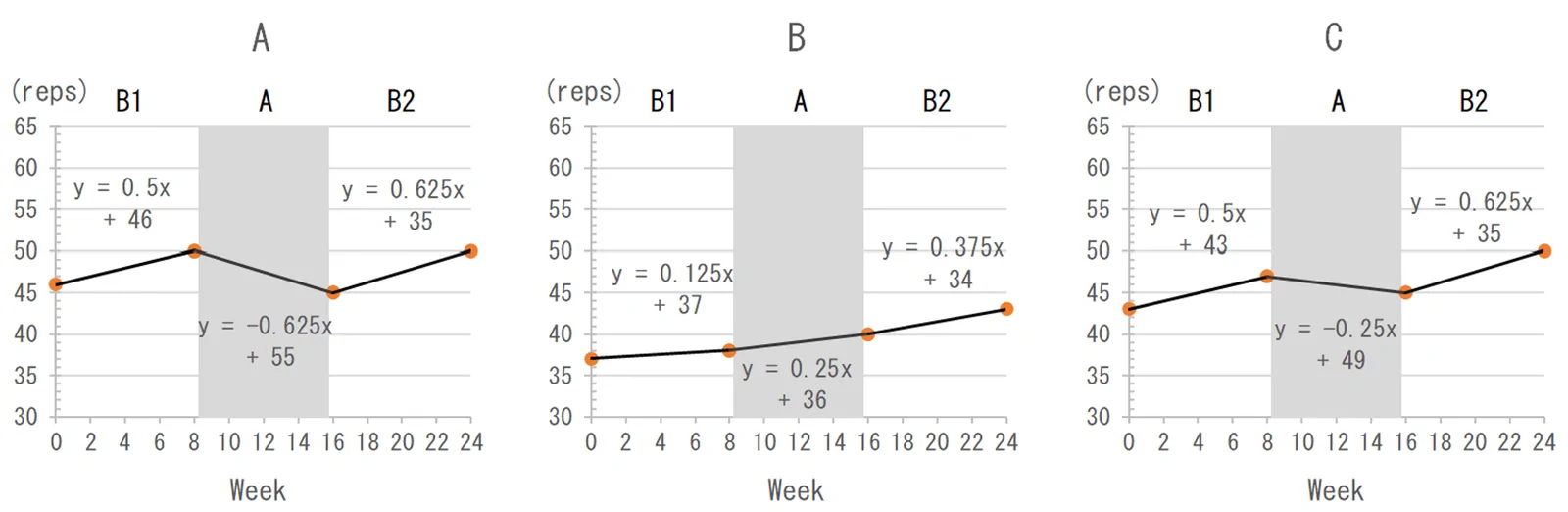
Sit-up test outcomes: participants A, B, and C Sit-up repetitions during the intervention period.

Sit-up performance improved in all participants over the intervention period. Participant A increased from 46 repetitions at baseline to 50 repetitions at the end of B2. Participant B improved from 37 to 43 repetitions, while Participant C improved from 43 to 50 repetitions. In all participants, performance tended to plateau or decline during the withdrawal phase before increasing again during the second intervention phase. Regression analysis showed the greatest positive slopes during B2, indicating that improvements were most pronounced following reintroduction of the intervention (Figure 4).

**Figure 5:**
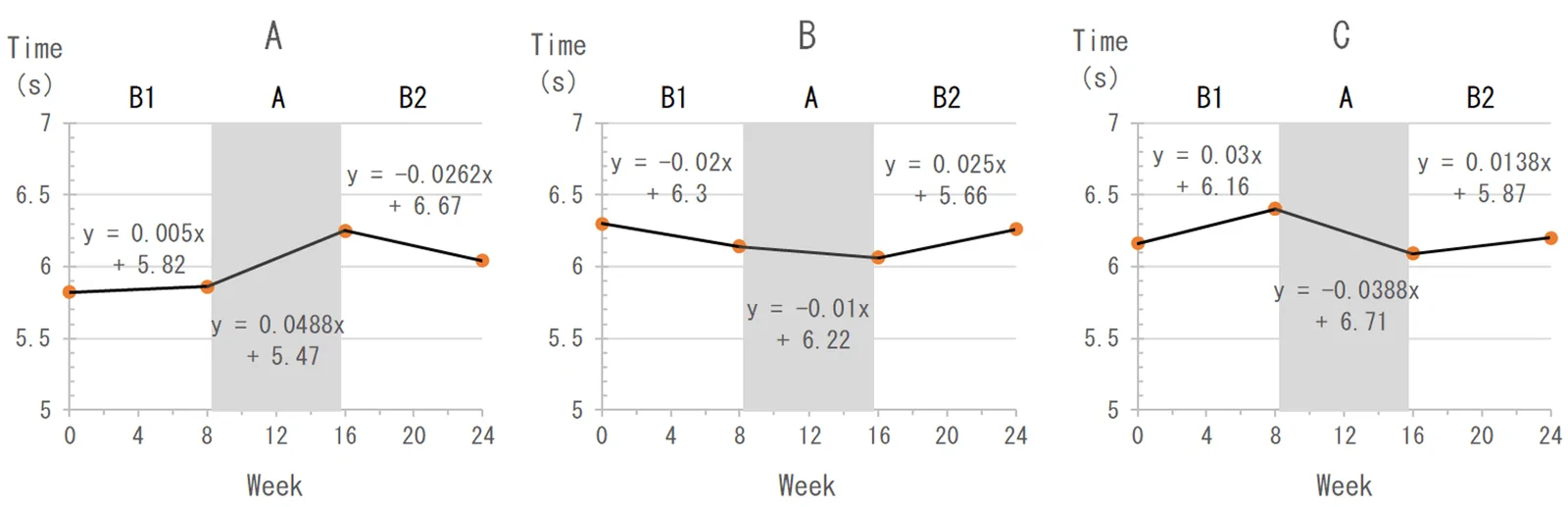
A 40-yard dash outcomes: participants A, B, and C Changes in 40-yard dash time during the intervention period.

The 40-yard dash showed little change throughout the study period. Sprint times ranged from 5.82 to 6.04 s for Participant A, from 6.30 to 6.26 s for Participant B, and from 6.16 to 6.20 s for Participant C, suggesting that the intervention had minimal influence on sprint performance (Figure 5).

Body Composition

Body fat percentage (PBF), body fat mass (BFM), and skeletal muscle mass (SMM) remained generally stable throughout the 24-week study period in all participants (Appendix 1).

Subjective Questionnaire Assessment

All participants reported that the program was easy to continue and did not interfere with their academic studies. Fatigue scores remained low throughout the intervention. Participant B expressed particularly strong interest in adopting additional exercises, whereas Participant C reported more moderate interest, suggesting individual differences in attitudes toward a future conditioning program (Table 2).

**Table 2:** Subjective questionnaire assessment All items were rated using a 5-point Likert scale (1 = strongly disagree, 5 = strongly agree). Higher scores reflect more favorable perceived improvements for items 1-6 and 8-12, whereas for items 7, 13, and 14, higher scores indicate greater ease of adherence or less interference with university life.

	1. Have you noticed any changes in your core stability?	2. Have you noticed any changes in your balance or stability during skating?	3. Have you noticed any changes in the strength of your abdominal or back muscles?	4. Since starting plank training, have you felt any changes in how easily you get tired after playing?	5. Have you noticed any changes in the quality of your performance (e.g., speed, agility, or posture control)?	6. Have you felt more stable during physical contact or when turning?	7. Was it easy to continue the 30-second plank training three times a week?	8. How did you feel about the intensity of the plank training?	9. Has this training changed the way you think about your body or muscle strength?	10. Has this training affected your motivation - for example, made you more likely to do other exercises on your own?	11. Would you like to incorporate the 30-second plank training into your future training routine?	12. Would you like to try other types of core training?	13. Did the training interfere with your university life, such as reducing time for assignments?	14. Did you experience any fatigue from the training that affected your university life, such as feeling sleepy during class?
A	3	3	4	3	3	3	5	4	4	4	4	5	5	5
B	4	4	4	2	4	4	5	3	4	4	5	5	4	5
C	3	3	4	3	3	3	5	4	4	4	3	4	5	5

## Discussion

The present prospective case series explored the feasibility and potential effects of a minimal-dose core training program in three elite female university ice hockey players. Although only three athletes were included, a consistent pattern emerged across the BAB phases: core function improved during both intervention periods, whereas these improvements plateaued or partially declined during the withdrawal phase. This repeated pattern strengthens the possibility that the observed changes were associated with the intervention rather than normal seasonal variation alone.

The greatest improvements were observed in measures of core function, particularly the SCST and the SST. Improvements in sit-up performance were also observed, whereas sprint performance and body composition remained relatively unchanged. These findings suggest that an extremely short-duration plank exercise may preferentially influence trunk motor control and muscular endurance rather than whole-body athletic performance [7,14].

One possible explanation is that repeated low-volume isometric exercise primarily promotes neuromuscular adaptation. Early improvements following resistance exercise are frequently attributed to enhanced motor unit recruitment, improved intermuscular coordination, and greater movement efficiency before measurable muscle hypertrophy occurs [6,8]. Because all participants were already elite athletes with many years of competitive experience, large structural adaptations would not be expected following such a brief intervention. Instead, improved trunk stabilization and motor control may explain the consistent changes observed in the SCST and SST [15,16]. Nevertheless, neuromuscular function was not directly measured in this study. Therefore, this interpretation should be regarded as a hypothesis rather than a confirmed mechanism.

Interestingly, the intervention appeared to affect different aspects of physical performance. Measures that depended primarily on trunk stability and muscular endurance improved, whereas the 40-yard dash showed little change. Sprint performance depends on multiple physiological factors, including lower-limb power, acceleration mechanics, skating-specific technique, and whole-body coordination [5]. Consequently, a single static trunk exercise alone would not be expected to substantially influence explosive sprint ability. This distinction suggests that minimal-dose core training should be considered a complementary conditioning strategy rather than a comprehensive performance enhancement program.

An important observation from this case series was the high level of adherence. All participants completed the intervention without interruption, reported that the program did not interfere with university life, and rated the exercise as easy to continue. These findings are particularly meaningful because university athletes often struggle to balance academic responsibilities with competitive sport. From a practical perspective, a conditioning program requiring only 30 seconds per session may be substantially easier to integrate into existing training schedules than conventional core exercise programs.

Another noteworthy finding was the discrepancy between objective and subjective outcomes. Although objective measures of core function consistently improved, participants reported only modest subjective changes in balance and core stability. Similar discrepancies have been described in sports medicine, where measurable improvements in motor performance are not always consciously perceived by athletes. This observation highlights the importance of combining objective performance testing with self-reported outcomes when evaluating athletic conditioning programs.

This study also illustrates the practical value of the BAB single-case experimental design in elite sports settings. Randomized controlled trials are often difficult to conduct in highly trained athletes because of limited sample availability, competitive schedules, and ethical concerns regarding withholding training [9]. Repeated within-participant observations allowed each athlete to serve as her own control and demonstrated consistent responses across intervention and withdrawal phases, thereby strengthening confidence in the observed temporal relationship.

Several limitations should be acknowledged. First, this case series included only three athletes from a single university team, limiting generalizability. Second, no direct physiological measurements of muscle activation or neuromuscular function were obtained, preventing confirmation of the proposed mechanisms. Third, body composition was assessed using bioelectrical impedance analysis, which may have lacked sufficient sensitivity to detect subtle changes in trunk musculature [17]. Finally, although the BAB design strengthened within-subject interpretation, causal inference remains limited compared with randomized controlled studies [9].

Despite these limitations, this study provides preliminary evidence that an extremely brief core training program can be successfully implemented in elite female university ice hockey players without disrupting academic or athletic activities. The consistent improvements observed across repeated intervention phases support the feasibility of this approach and provide proof-of-concept for future larger-scale investigations. These findings are particularly meaningful in the context of dual-career athletes, who frequently experience difficulties balancing academic responsibilities and athletic participation.

## Conclusions

This prospective case series suggests that a minimal-dose core training program consisting of a single 30-second prone plank performed three times per week may improve core function and trunk muscular endurance in elite female university ice hockey players while imposing minimal time burden. Improvements were observed primarily in measures of trunk motor control rather than sprint performance or body composition, suggesting that neuromuscular adaptation may be the principal mechanism.

Although the findings cannot be generalized because of the very small sample size, the intervention demonstrated excellent feasibility, adherence, and acceptability among university athletes. These results support further prospective controlled studies to determine whether similarly time-efficient conditioning strategies can enhance athletic performance in larger athletic populations.

## Appendices

Appendix 1

**Table 3:** Body composition results of participants A, B, and C PBF: Percent Body Fat; BFM: Body Fat Mass; SLM: Soft Lean Mass; SMM: Skeletal Muscle Mass

	Time (week)	A					B					C				
		Body weight (kg)	BFM (kg)	PBF (%)	SLM (kg)	SMM (kg)	Body weight (kg)	BFM (kg)	PBF (%)	SLM (kg)	SMM (kg)	Body weight (kg)	BFM (kg)	PBF (%)	SLM (kg)	SMM (kg)
B1	0	61	13.3	21.9	44.9	26.3	58.8	15.9	27	40.4	23.8	60.9	16.9	27.7	41.5	24.1
	4	60.8	12.6	20.7	45.4	26.7	58.5	15.5	26.5	40.5	23.8	60.3	17.1	28.4	40.8	23.5
A	8	60.3	12.2	20.2	45.3	26.6	59.3	15.7	26.5	41	24.1	57.3	14.5	25.3	40.4	23.4
	12	-	-	-	-	-	-	-	-	-	-	-	-	-	-	-
	16	59.9	12.9	21.5	44.3	26	59.8	18.2	30.4	39.2	23.1	60.4	15.8	26.1	42.1	24.7
B2	20	59.9	12.4	20.7	44.8	26.2	-	-	-	-	-	-	-	-	-	-
	24	60.7	12	19.8	45.9	26.9	59.6	16.8	28.2	40.3	23.6	63.2	18.3	28.9	42.4	24.7

**Table 4:** Segmental muscle mass results of participants A, B, and C

	Time (week)	A					B					C				
		Right upper limb (kg)	Left upper limb (kg)	Trunk (kg)	Right lower limb (kg)	Left Lower limb (kg)	Right upper limb (kg)	Left upper limb (kg)	Trunk (kg)	Right lower limb (kg)	Left Lower limb (kg)	Right upper limb (kg)	Left upper limb (kg)	Trunk (kg)	Right lower limb (kg)	Left Lower limb (kg)
B1	0	2.32	2.33	20.6	7.89	7.78	2.07	2.09	18.7	6.52	6.44	2.21	2.26	19.6	6.52	6.44
	4	2.41	2.4	21.1	7.59	7.6	2.02	2.08	18.6	6.52	6.46	2.15	2.13	19	6.52	6.46
A	8	2.4	2.38	20.9	7.74	7.74	2.08	2.13	18.8	6.66	6.51	2.04	2.12	18.7	6.66	6.51
	12	-	-	-	-	-	-	-	-	-	-	-	-	-	-	-
	16	2.3	2.28	20.5	7.59	7.6	2.02	1.98	18.4	6.42	6.41	2.19	2.26	19.6	6.42	6.41
B2	20	2.27	2.28	20.3	7.85	7.93	-	-	-	-	-	-	-	-	-	-
	24	2.3	2.31	20.4	8.06	8.15	1.98	2	18.3	6.63	6.76	2.19	2.24	19.5	6.63	6.76
